# A real-world study and network pharmacology analysis of EGFR-TKIs combined with ZLJT to delay drug resistance in advanced lung adenocarcinoma

**DOI:** 10.1186/s12906-023-04213-3

**Published:** 2023-11-21

**Authors:** Xue Han, Lan Liang, Chenming He, Qinyou Ren, Jialin Su, Liang Cao, Jin Zheng

**Affiliations:** 1 Shaanxi University of Chinese Medicine, Shiji Avenue, Xixian new area, Xianyang, Shaanxi China; 2 The Second Affiliated Hospital of Air Force Medical University, Xinsi Avenue, Baqiao Area, Xi’an, Shaanxi China

**Keywords:** Lung adenocarcinoma, ZiLongJin Tablet, Epidermal growth factor receptor-tyrosine kinase inhibitor, Real-world study, Network pharmacology, Molecular docking simulation

## Abstract

**Objective:**

This study aimed to explore the efficacy and safety of combining epidermal growth factor receptor tyrosine kinase inhibitors (EGFR-TKIs) with ZiLongJin Tablet (ZLJT) in delaying acquired resistance in advanced EGFR-mutant lung adenocarcinoma (LUAD) patients. Furthermore, we employed network pharmacology and molecular docking techniques to investigate the underlying mechanisms.

**Methods:**

A retrospective comparative study was conducted on stage IIIc/IV LUAD patients treated with EGFR-TKIs alone or in combination with ZLJT at the Second Affiliated Hospital of the Air Force Medical University between January 1, 2017, and May 1, 2023. The study evaluated the onset of TKI resistance, adverse reaction rates, safety indicators (such as aspartate aminotransferase, alanine aminotransferase, and creatinine), and inflammatory markers (neutrophil-to-lymphocyte ratio and platelet-to-lymphocyte ratio) to investigate the impact of EGFR-TKI combined with ZLJT on acquired resistance and prognostic indicators. Additionally, we utilized the Traditional Chinese Medicine Systems Pharmacology Database and Analysis Platform, the Bioinformatics Analysis Tool for Molecular Mechanism of Traditional Chinese Medicine, PubChem, UniProt, and Swiss Target Prediction databases to identify the active ingredients and targets of ZLJT. We obtained differentially expressed genes related to EGFR-TKI sensitivity and resistance from the Gene Expression Omnibus database using the GSE34228 dataset, which included sensitive (*n* = 26) and resistant (*n *= 26) PC9 cell lines. The "limma" package in R software was employed to detect DEGs. Based on this, we constructed a protein‒protein interaction network, performed gene ontology and KEGG enrichment analyses, and conducted pathway network analysis to elucidate the correlation between the active ingredients in ZLJT and signaling pathways. Finally, molecular docking was performed using AutoDockVina, PYMOL 2.2.0, and Discovery Studio Client v19.1.0 software to simulate spatial and energy matching during the recognition process between predicted targets and their corresponding compounds.

**Results:**

(1) A total of 89 patients were included, with 40 patients in the EGFR-TKI combined with ZLJT group (combination group) and 49 patients in the EGFR-TKI alone group (monotherapy group). The baseline characteristics of the two groups were comparable. There was a significant difference in the onset of resistance between the combination group and the monotherapy group (*P* < 0.01). Compared to the monotherapy group, the combination group showed a prolongation of 3.27 months in delayed acquired resistance. There was also a statistically significant difference in the onset of resistance to first-generation TKIs between the two groups (*P* < 0.05). (2) In terms of safety analysis, the incidence of adverse reactions related to EGFR-TKIs was 12.5% in the combination group and 14.3% in the monotherapy group, but this difference was not statistically significant (*P* > 0.05). There were no statistically significant differences in serum AST, ALT, CREA, TBIL, ALB and BUN levels between the two groups after medication (*P* > 0.05). (3) Regarding inflammatory markers, there were no statistically significant differences in the changes in neutrophil-to-lymphocyte Ratio(NLR) and Platelet-to-lymphocyte Ratio(PLR) values before and after treatment between the two groups (*P* > 0.05). (4) Network pharmacology analysis identified 112 active ingredients and 290 target genes for ZLJT. From the GEO database, 2035 differentially expressed genes related to resistant LUAD were selected, and 39 target genes were obtained by taking the intersection. A "ZLJT-compound-target-disease" network was successfully constructed using Cytoscape 3.7.0. GO enrichment analysis revealed that ZLJT mainly affected biological processes such as adenylate cyclase-modulating G protein-coupled receptor. In terms of cellular components, ZLJT was associated with the cell projection membrane. The molecular function primarily focused on protein heterodimerization activity. KEGG enrichment analysis indicated that ZLJT exerted its antitumor and anti-drug resistance effects through pathways such as the PI3K-Akt pathway. Molecular docking showed that luteolin had good binding activity with FOS (-9.8 kJ/mol), as did tanshinone IIA with FOS (-9.8 kJ/mol) and quercetin with FOS (-8.7 kJ/mol).

**Conclusion:**

ZLJT has potential antitumor progression effects. For patients with EGFR gene-mutated non-small cell LUAD, combining ZLJT with EGFR-TKI treatment can delay the occurrence of acquired resistance. The underlying mechanisms may involve altering signal transduction pathways, blocking the tumor cell cycle, inhibiting tumor activity, enhancing cellular vitality, and improving the bioavailability of combination therapy. The combination of EGFR-TKI and ZLJT represents an effective approach for the treatment of tumors using both Chinese and Western medicine.

## Highlights

1. Combining EGFR-TKIs with ZLJT showed a significant prolongation in the onset of resistance in advanced EGFR-mutant lung adenocarcinoma patients.

2. Combination therapy did not increase the incidence of adverse reactions compared to EGFR-TKI monotherapy.

3. ZLJT targeted key signaling pathways, such as PI3K-Akt, which are associated with tumor progression and drug resistance.

4. Molecular docking analysis revealed strong binding activity between ZLJT active ingredients (luteolin, tanshinone IIA, and quercetin) and the FOS protein, indicating their potential role in delaying acquired resistance.

5. Combining ZLJT with EGFR-TKIs may alter signal transduction pathways, block the tumor cell cycle, inhibit tumor activity, enhance cellular vitality, and improve treatment effectiveness.

6. The combination of Western and Chinese medicine using EGFR-TKI and ZLJT represents a promising tumor treatment strategy.

## Introduction

Lung cancer is the most common malignant tumor in 36 countries, including China. It ranks first in terms of cancer incidence among males and third among females in China [1]. Non-small cell lung carcinoma (NSCLC) accounts for approximately 85% of all lung cancers. Among them, LUAD represents approximately 50% of NSCLC cases [2]. Currently, LUAD is classified into molecular subgroups based on genetic alterations, including those of EGFR, KRAS, ALK, ROS1, and BRAF. Among these, EGFR gene-mutated LUAD is the most common form of adenocarcinoma in East Asia; it accounts for 47%-62% of LUAD cases [3–5].

According to the 2021 NCCN guidelines for non-small cell lung cancer (NSCLC), osimertinib has become the recommended adjuvant therapy for stage Ib-IIIa NSCLC patients with EGFR mutations after surgery [6]. This marks the entry of EGFR-targeted therapy into adjuvant treatment for early-stage lung cancer. For stage IV NSCLC patients with sensitive EGFR mutations, first-line treatment options with a level I recommendation include EGFR tyrosine kinase inhibitors (TKIs), including first-, second-, and third-generation TKIs. For patients with oligoprogressive or central nervous system progression, the level I recommendation is to continue the original TKI treatment along with local therapy. In cases of widespread metastasis and treatment failure with first- or second-generation TKIs, rebiopsy is recommended. For patients with T790M-positive mutations, third-generation TKIs, such as osimertinib, furmonertinib, or almonertinib, are recommended. Osimertinib, compared to previous generations, irreversibly binds to mutant forms of EGFR (T790M, L858R, and exon 19 deletion), primarily targeting the kinase domain of EGFR. It competitively binds to the ATP-binding site on the kinase, inhibiting EGFR phosphorylation and blocking downstream signaling pathway activation. It exhibits higher selectivity for mutant EGFR over wild-type EGFR [7, 8]. EGFR-TKIs hold a significant position in the treatment of NSCLC patients [9]. Studies have demonstrated significant benefits of EGFR-TKIs, with a median progression-free survival (PFS) of approximately 9–13 months. However, acquired resistance ultimately develops. Currently, there are no effective clinical measures to address acquired resistance [10]. A more comprehensive exploration of clinical research on acquired resistance is crucial for developing more effective treatment strategies.

In recent years, traditional Chinese medicine (TCM) has shown significant efficacy in the treatment of lung cancer progression and the inhibition of drug resistance [11, 12]. Among these TCMs, ZLJT (Approval Number Z20010064) is included in the 2015 edition of the Chinese Pharmacopoeia Part I. It is a compound formulation developed based on the theory of TCM. The main ingredients of ZLJT include Huangqi (*Astragalus membranaceus*), Danggui (*Angelica sinensis*), Baiying (*Solanum lyratum Thunb.*), Longkui (*Solanum nigrum L.*), Danshen (*Salvia miltiorrhiza*), Shemei (*Duchesnea indica*), Banzhilian (*Scutellaria barbata*), and Yujin (*Curcuma wenyujin/Curcuma Longa/Curcuma kwangsiensis/Curcuma phaeocaulis*). Studies have shown that ZLJT has inhibitory effects on tumor occurrence and development, improves hematopoietic function, and enhances the body's immune system. It is widely used in patients with advanced-stage non-small cell lung cancer (NSCLC) [13]. Huangqi in the formulation can regulate macrophage polarization and synergistically enhance the anticancer effects of chemotherapy drugs [14]. Huangqi saponin IV, one of the main active components of Huangqi, can increase the sensitivity of tumor cells to gefitinib by modulating SIRT6 [15]. Danggui has been proven to suppress lung cancer progression, cancer cachexia, and cancer-related inflammation through its immunomodulatory functions [16]. Baiying extract can enhance NK cell activity and increase the number of CD4 cells in tumor-bearing mice, significantly improving their survival rate [17]. Longkui significantly increases downstream ROS production in a dose-dependent manner. ROS induce cell cycle arrest, senescence, and apoptosis in cancer cells through lipid peroxidation [18]. Compounds in Danshen, such as cryptotanshinone (CTN), exhibit various biological activities, including antithrombotic, anti-inflammatory, and antitumor effects. CTN in the methanol extract of Danshen induces cell apoptosis through mitochondrial pathways and inhibits the PI3K/Akt pathway mediated by PTEN, effectively suppressing tumor growth [19]. Shemei inhibits cell growth. It reduces MMP-2 protein expression and MMP-2 activity in a dose-dependent manner and decreases phosphorylation of ERK1/2 and its upstream kinases, thereby increasing antitumor metastasis activity [20]. Banzhilian can induce cytotoxicity by regulating multiple apoptotic pathways in different NSCLC cells [21]. Yujin has antitumor and antimetastatic activities. It inhibits cell proliferation and metastasis by regulating the expression of metastasis markers, including C–C chemokine receptor type 7, matrix metalloproteinase 9, and the proto-oncogenes c-fos and c-jun [22].

This study aims to evaluate the efficacy and safety of combining EGFR-TKIs with ZLJT in delaying acquired resistance. Furthermore, it aims to explore the underlying mechanisms using network pharmacology and molecular docking techniques.

## Materials and methods

### Clinical data acquisition

#### Source of case data

All patients included in this study were recruited from the Second Affiliated Hospital of Air Force Medical University. They were diagnosed with LUAD and received EGFR-TKIs with or without ZLJT from January 1, 2017, to May 1, 2023. Drug resistance occurred during this period.

#### Patient selection

Inclusion Criteria were as follows: diagnosed with stage IIIc or stage IV LUAD based on pathological or cytological examination; pathological tissue specimens confirmed through real-time quantitative PCR to have an EGFR gene exon 19 deletion mutation (19Del) or exon 21 L858R mutation (L858R); receiving EGFR-TKI treatment for the first time, and the combination group should start taking ZLJT (7.8 g/day) concurrently with the first dose of EGFR-TKI and continue for at least 8 weeks; and aged 18 years or older without a history of significant heart, lung, liver, kidney, blood system, or other severe diseases. To exclude interference from primary resistance, patients will be re-evaluated within 8 weeks of TKI initiation to confirm no disease progression.

Exclusion Criteria were as follows: failure to meet any of the inclusion criteria; presence of severe cardiovascular, cerebrovascular, or mental disorders; and insufficient medical records or data for analysis.

### Drug treatment regimen

#### EGFR-TKI treatment regimen

First-generation TKI drugs: a. gefitinib tablets: 250 mg once daily, orally, on an empty stomach or with food until disease progression, intolerable toxic side effects, or patient request to discontinue; b. erlotinib tablets: 150 mg once daily, orally, on an empty stomach or with food until disease progression, intolerable toxic side effects, or patient request to discontinue; and c. icotinib tablets: 125 mg three times daily, orally, on an empty stomach or with food until disease progression, intolerable toxic side effects, or patient request to discontinue.

Second-generation TKI drugs: a. afatinib tablets: 40 mg once daily, orally, on an empty stomach or with food until disease progression, intolerable toxic side effects, or patient request to discontinue; and b. dacomitinib tablets: 45 mg once daily, orally, on an empty stomach or with food until disease progression, intolerable toxic side effects, or patient request to discontinue.

Third-generation TKI drugs: a. osimertinib tablets: 80 mg once daily, orally, on an empty stomach or with food until disease progression, intolerable toxic side effects, or patient request to discontinue; b. furmonertinib tablets: 80 mg once daily, orally, on an empty stomach or with food until disease progression, intolerable toxic side effects, or patient request to discontinue; and c. almonertinib tablets: 110 mg once daily, orally, on an empty stomach or with food until disease progression, intolerable toxic side effects, or patient request to discontinue.

ZLJT treatment regimen: 2.6 g per dose (0.65 g per tablet), three times daily, orally, with a treatment course of every 56 days.

### Research methods

#### Clinical observation indicators

Primary Outcome Measures: The main outcome measure is the time from the initiation of EGFR-TKIs combined with ZLJT or EGFR-TKIs alone to the occurrence of disease progression. The difference in time represents the development of acquired resistance.

Secondary Outcome Measures: The incidence of EGFR-TKI-related adverse events will be recorded. If a patient experiences any adverse reaction related to TKI medication during the treatment period, it will be counted as 1; otherwise, it will be recorded as 0. Changes in liver and kidney function before and after treatment will be monitored in both groups. AST, ALT, CREA, TBIL, ALB and BUN will be used as safety indicators. A comparison of changes in immune and inflammatory markers before and after TKI treatment will be conducted between the two groups.

#### Clinical data collection methods

A retrospective case data research form was prepared and used to collect information on all enrolled cases. The form includes the following details: basic information of the enrolled cases, such as name, sex, and age; disease-related information, such as tumor location, pathological type, and clinical staging; and treatment history prior to enrollment, including whether surgery was performed or not. It is important to ensure objective and accurate completion of the form. In case of any errors, corrections were made by crossing out the incorrect information with a horizontal line and providing the correct information next to it. The corrections were dated and signed by the person making the modifications.

#### ZLJT active ingredient screening and target prediction

We used TCMSP (https://tcmsp-e.com/) and BATMAN-TCM (http://bionet.ncpsb.org.cn/batman-tcm/) to search for the active ingredients of "Huangqi", "Yujin", "Danggui", "Shemei", "Baiying", "Banzhilian", "Longkui", and "Danshen" in ZLJT. In TCMSP, we used the conditions of oral bioavailability (OB) ≥ 30% and drug likeness (DL) ≥ 0.18 for screening. Since the TCMSP database did not include "Shemei," we used BATMAN-TCM. We set the cutoff value of the input parameter score as ≥ 20 and adjusted *P* < 0.05 to obtain the active ingredients and targets of "Shemei." The compounds were standardized into simplified molecular inputs and converted into SMILES format, which were then submitted to the Swiss Target Prediction Database (http://www.swisstargetprediction.ch/). By selecting "Homo sapiens" as the filtering condition, the potential therapeutic targets of ZLJT active ingredients were predicted. The compound-target interactions were standardized using the UniProt database (https://www.uniprot.org).

#### Differentially Expressed Genes (DEGs) in EGFR-TKI-sensitive and -resistant LUAD cells

DEGs of EGFR-TKI-sensitive and -resistant cells were obtained from the GEO database (https://www.ncbi.nlm.nih.gov/geo/). The GSE34228 dataset includes 26 PC9 cell lines classified as either sensitive or resistant to gefitinib. The Limma package in R version 4.3.0 was utilized for integrated analysis of multiple microarrays, and proper data batch processing was performed (https://www.bioconductor.org/). DEGs were selected based on an adjusted *P* < 0.05 and log2-fold change > 1 or log2-fold change < -1 [23, 24].

#### Integrating common targets between ZLJT and DEGs

The intersection of targets between ZLJT active ingredients and DEGs was found using R 4.3.0 Language software.

#### Constructing a "ZLJT-compound-target-disease" regulatory network

A "ZLJT-compound-target-disease" regulatory network was constructed in Cytoscape 3.7.0 software.

#### Constructing a Protein‒Protein Interaction (PPI) network

The obtained intersecting targets were imported into the STRING database (https://string-db.org/) to construct a PPI network. The species was limited to Homo sapiens (human), with a medium confidence greater than 0.9, and discrete targets were excluded. The PPI network graph was generated in TSV format for download. The file was imported into Cytoscape software to build the PPI network, and the degree algorithm was used to calculate the top 20 core targets and export them.

### Statistical analysis methods

#### Statistical analysis of clinical data

The obtained clinical data were input into a computer, and a relevant database was established. EmpowerStats software (http://www.empowerstats.com) was used for statistical analysis. For normally distributed continuous data with homogeneous variances, t tests were used for group comparisons. For continuous data that did not follow a normal distribution, the median (interquartile range) was used, and nonparametric tests, such as the Mann‒Whitney U test, were performed. For categorical data, percentages are used, and within-group comparisons are conducted using the chi-square test or, if necessary, Fisher's exact test. *P* < 0.05 was considered to indicate statistically significant.

#### Network pharmacology analysis

To perform network pharmacology analysis, the following steps were taken. First, using the org.Hs.eg.db package in R version 4.3.0, the gene names of the intersecting targets were converted to Entrez IDs. Next, the ClusterProfiler package in R version 4.3.0 was used for functional enrichment analysis, including Gene Ontology (GO) enrichment analysis for molecular functions (MFs), biological processes (BPs), and cellular components (CCs), and KEGG pathway enrichment analysis was performed to elucidate the pathways involved in the inhibition of EGFR-TKIs resistance in LUAD by ZLJT. The results of GO enrichment and KEGG pathway enrichment were visualized and presented using bubble plots [25].

#### Molecular docking simulation

**Fig. 1:**
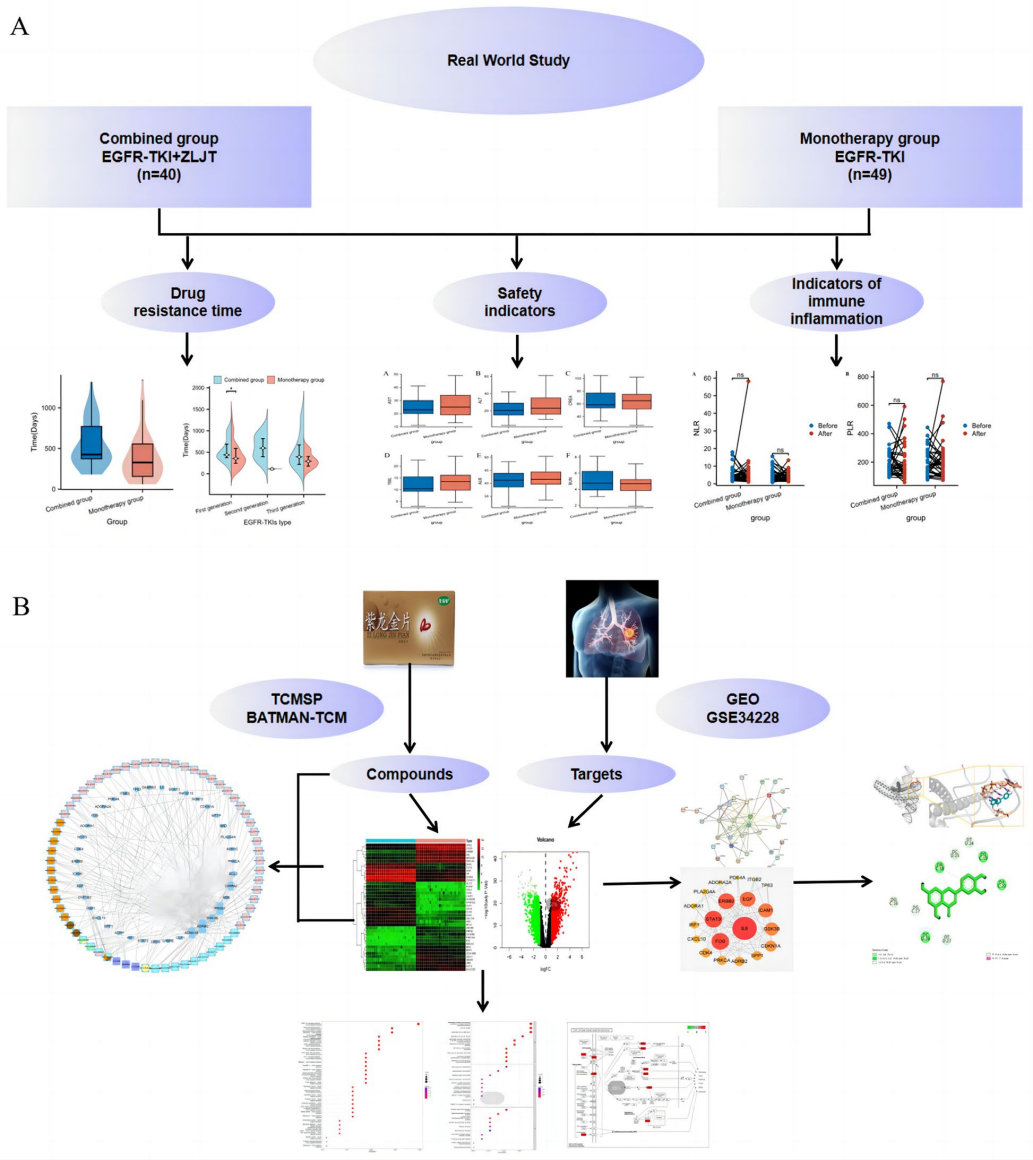
The clinical and network pharmacology process of ZLJT in delaying drug resistance in advanced lung adenocarcinoma. **A** Clinical research findings. **B** Network pharmacology and molecular docking

Molecular docking was performed with the main active compounds in ZLJT and the core target proteins. Protein crystal structures were obtained from the PDB database (https://www.rcsb.org/), and the chemical structures of compounds were obtained from the PubChem database (https://pubchem.ncbi.nlm.nih.gov/). The small molecule structures (in PDB format) were drawn using ChemDraw 8.0.3 for docking. Autodock 4.2.6 was used for virtual docking between the compounds and protein targets, with only the ligand conformation changed while keeping the protein conformation unchanged. All other parameters were set to default values. The docking results were visualized and analyzed using PyMOL 2.2.0 and Discovery Studio Client v19.1.0 software. The research process is illustrated in Fig. 1.

## Results

### General patient characteristics

As of May 2023, a total of 89 advanced-stage LUAD patients from the Second Affiliated Hospital of Air Force Medical University were included. Among them, 61 were male, and 28 were female. The median age was 57 years. Out of all patients, 26 had undergone surgery. According to the revised Lung Cancer International Staging Manual by the American Cancer Society [26], 14 patients were in stage IIIc, 57 patients were in stage IVa, and 18 patients were in stage IVb. The primary tumor was distributed throughout the lung. Among the included patients, there were 40 patients in the combination group. This group included 31 males and 9 females, and the average age was 59.25 ± 12.24 years. Among them, 11 patients underwent surgery, including 8 in stage IIIc, 25 in stage IVa, and 7 in stage IVb. TKI drug use in the combination group included first-generation drugs (gefitinib, erlotinib, and icotinib) in 28 cases, second-generation drugs (afatinib and dacomitinib) in 4 cases, and third-generation drugs (osimertinib, furmonertinib, and almonertinib) in 8 cases. There were 49 patients in the monotherapy group. This group included 30 males and 19 females, and the average age was 56.53 ± 8.57 years. Among them, 15 patients underwent surgery, including 6 in stage IIIc, 32 in stage IVa, and 11 in stage IVb. TKI drug use in the monotherapy group included first-generation drugs in 34 cases, second-generation drugs in 2 cases, and third-generation drugs in 13 cases. There were no statistically significant differences between the two groups in terms of age, sex, presence of surgery, tumor stage and location, TKI drug use, and AST, ALT, CREA, TBIL, ALB, BUN levels (*P* > 0.05) (Table 1).

**Table 1:** Baseline characteristics comparison of the two groups

Characteristics	Combined group	Monotherapy group	t/c^2^/Z	*P*
n	40	49		
Age (years)	59.25 ± 12.24	56.53 ± 8.57	1.19	0.24
Sex			2.71	0.1
Males	31 (77.5%)	30 (61.2%)		
Females	9 (22.5%)	19 (38.8%)		
Surgery or not			0.1	0.75
Yes	11 (27.5%)	15 (30.6%)		
No	29 (72.5%)	34 (69.4%)		
Staging			1.14	0.57
IIIc	8 (20.0%)	6 (12.2%)		
Iva	25 (62.5%)	32 (65.3%)		
Ivb	7 (17.5%)	11 (22.4%)		
Tumor location			2.37	0.67
Right upper lobe	12 (30.0%)	18 (36.7%)		
Right middle lobe	7 (17.5%)	7 (14.3%)		
Right lower lobe	8 (20.0%)	5 (10.2%)		
Left upper lobe	8 (20.0%)	10 (20.4%)		
Left lower lobe	5 (12.5%)	9 (18.4%)		
EGFR-TKI type			1.52	0.52
First generation	28 (70.0%)	34 (69.4%)		
Second generation	4 (10.0%)	2 (4.1%)		
Third generation	8 (20.0%)	13 (26.5%)		
AST(U/L)	25.5 (20.0, 39.0)	27.0 (18.5, 37.0)	-0.82	0.41
ALT(U/L)	22.5 (17.3, 49.0)	28.0 (17.0, 43.0)	-0.042	0.68
CREA(μmoI/L)	58.0 (54.8, 71.0)	59.5 (48.7, 67.0)	-0.66	0.51
TBIL(μmoI/L)	11.3 (9.7, 13.2)	13.0 (9.4, 16.1)	-1.09	0.27
ALB(g/L)	40.5 (37.0, 44.5)	40.4 (37.2, 44.1)	-0.11	0.92
BUN(mmol/L)	4.6 (3.9, 5.6)	4.8 (3.9, 5.7)	-0.37	0.71

Data are mean (mean ± SD), median (IQR), n (%)

### Therapeutic evaluation

**Fig. 2:**
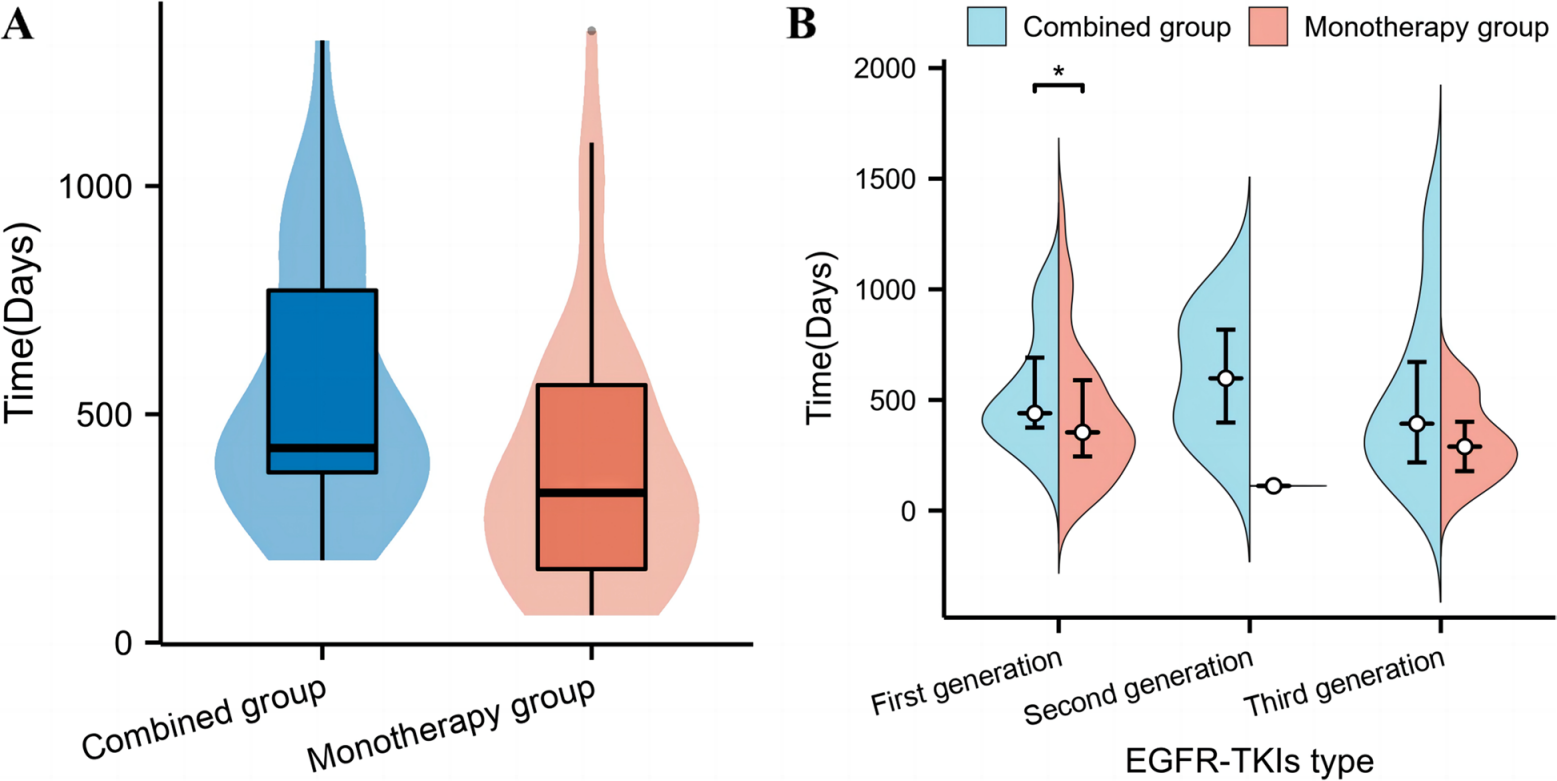
Comparison of the time to onset of acquired resistance. **A** Participants with or without ZLJT treatment. **B** Participants with different generations of drugs in the combination group (*P < 0.05)

The median time to acquired resistance was compared between the two groups. In the combination group, the median time to acquired resistance was 426.00 days (14.20 months), while in the monotherapy group, it was 328.00 days (10.93 months). Compared to the monotherapy group, the combination group showed a significantly longer time to acquired resistance, with an extension of 3.27 months (*P* < 0.01). There was also a significant difference in the onset of resistance to first-generation TKIs between the two groups (*P* < 0.05) (Table 2 and Fig. 2).

**Table 2:** Comparison of the time to onset of acquired resistance

	Combined group	Monotherapy group	Z	*P*
Total time of resistance onset (days)	426.00 (372.25, 773.75)	328.00 (158.00, 568.50)	-3.06	0.01
First generation drug resistance (days)	439.50 (374.25, 743.75)	353.50 (218.00, 598.00)	-2.06	0.04
Second generation drug resistance (days)	597.50 (363.25, 892.50)	111.50 (111.00, 112.00)	-1.85	0.06
Third generation drug resistance (days)	392.50 (212.50, 810.50)	289.00 (166.50, 466.00)	-1.27	0.21

Data are mean median (IQR)

### Safety indicators

**Fig. 3:**
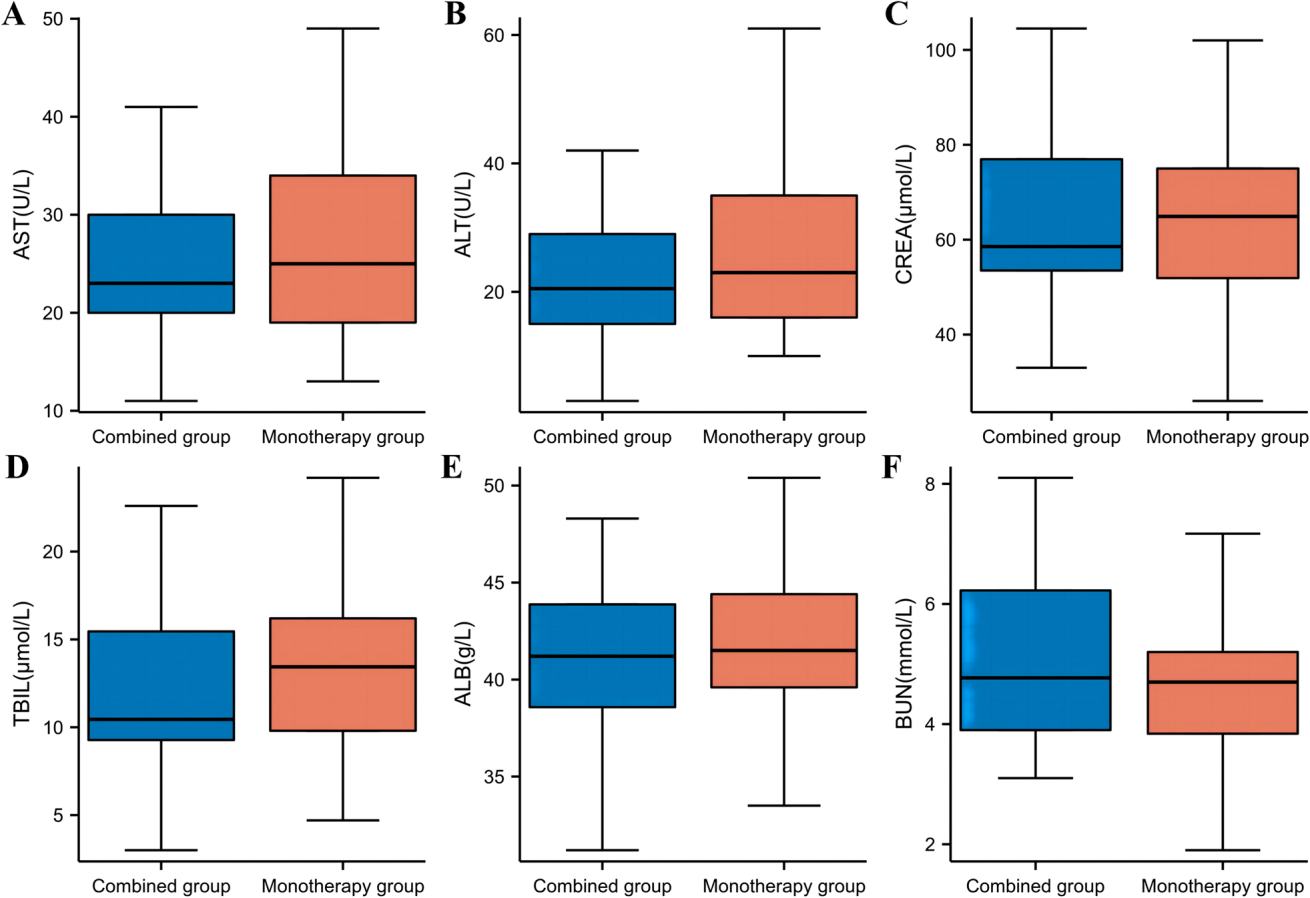
Comparison of serum AST, ALT, CREA, TBIL, ALB and BUN levels between the two patient groups. **A** AST, **B** ALT, **C** CREA, **D**TBIL, **E** ALB and **F** BUN

The incidence of adverse reactions related to EGFR-TKIs in the combination group and the monotherapy group was 12.5% and 14.3%, respectively (*P* > 0.05) (Table 3). The comparison of serum AST, ALT, CREA, TBIL, ALB and BUN levels between the two patient groups after medication showed *P* > 0.05, indicating no statistically significant differences (Table 4 and Fig. 3).

**Table 3:** Comparison of incidence rates of adverse reactions between the two patient groups

Adverse Reactions	Combination Group	Monotherapy Group	c^2^	*P*
Y	5 (12.5%)	7 (14.3%)	0.06	0.81
N	35 (87.5%)	42 (85.7%)		

Data are mean n (%)

**Table 4:** Comparison of serum AST, ALT, CREA, TBIL, ALB and BUN levels between the two patient groups

	Combination Group	Monotherapy Group	Z	*P*
AST(U/L)	23.0 (20.0, 30.0)	25.0 (19.0, 35.5)	-0.49	0.63
ALT(U/L)	20.5 (15.0, 29.0)	23.0 (16.0, 37.5)	-1.09	0.27
CREA(μmoI/L)	58.6 (52.5, 78.9)	64.9 (51.0, 75.0)	-0.26	0.79
TBIL(μmoI/L)	10.5 (9.3, 15.6)	13.4 (9.8, 16.2)	-1.11	0.27
ALB(g/L)	41.2 (38.6, 43.9)	41.5 (39.6, 44.4)	-0.55	0.58
BUN(mmoI/L)	4.8 (3.9, 6.5)	4.7 (3.8, 5.2)	-0.92	0.36

Data are mean median (IQR)

### Immunoinflammatory indicators

**Fig. 4:**
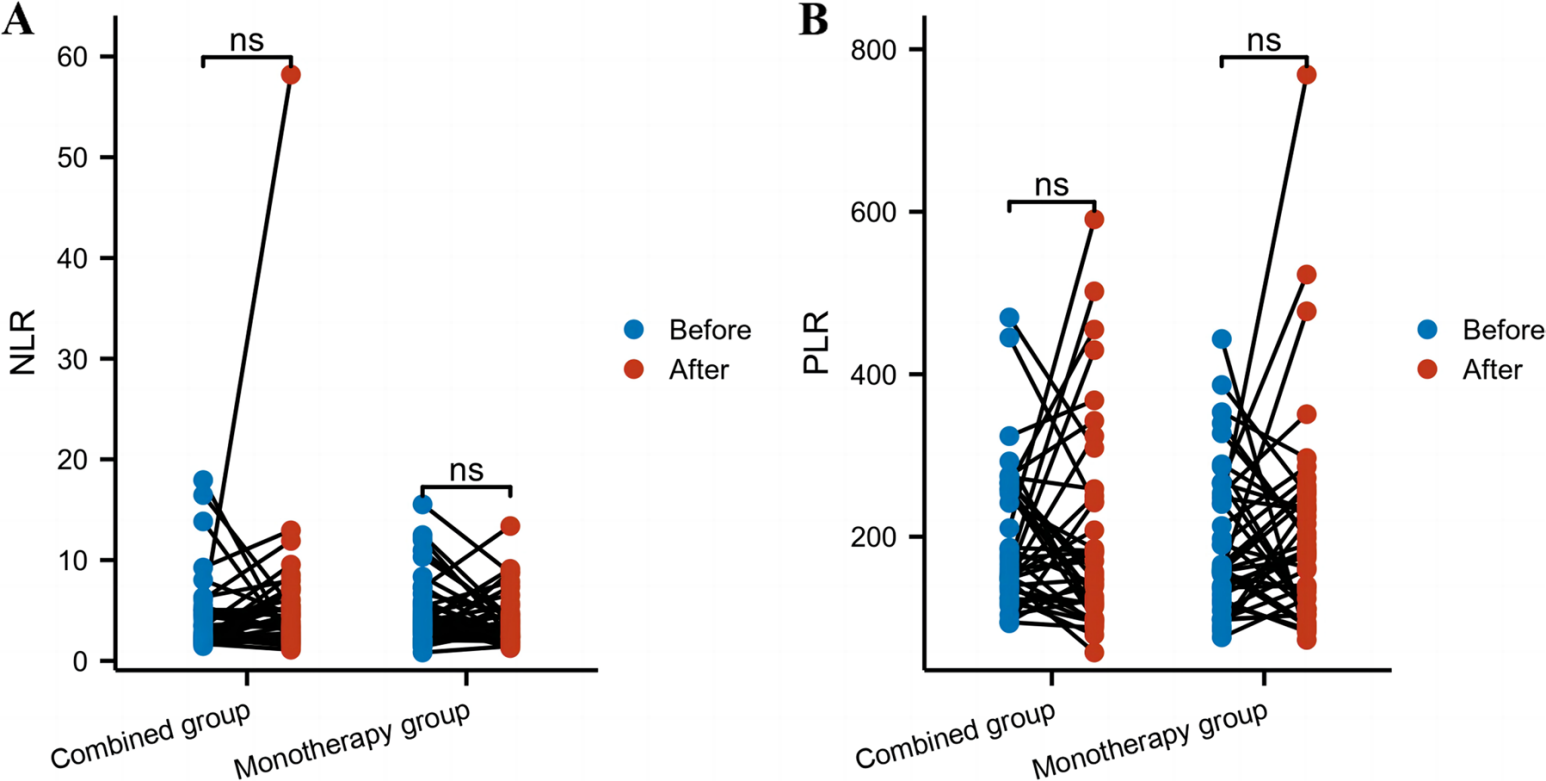
Comparison of the NLR and PLR between the two patient groups. **A** NLR and **B** PLR

The comparison of the neutrophil-to-lymphocyte ratio (NRL) and platelet-to-lymphocyte ratio (PLR) between the two groups before and after medication showed *P* > 0.05, indicating no statistically significant differences (Table 5 and Fig. 4).

**Table 5:** Comparison of the NLR and PLR between the two patient groups

		Combined group	Monotherapy group	Difference	95%CI	*P*
NLR	Before	3.4	3.6	0.55	-1.79	0.2
		(2.1, 5.0)	(2.7, 5.8)			
	After	3.4	3.2	-0.59	-2.12	0.15
		(2.4, 5.4)	(2.4, 4.6)			
PLR	Before	166.1	159.3	-14.63	-61.79	0.31
		(137.3, 258.7)	(132.2, 265.8)			
	After	153	180	-7.02	-70.27	0.7
		(114.0, 243.0)	(111.7, 249.1)			

Data are mean median (IQR)

### ZLJT active ingredient screening and target prediction

By searching the TCMSP and BATMAN databases, a total of 144 active ingredients that met the criteria were obtained in ZLJT (Table 6 and Table 7). Among them, there were 20 Huangqi, 2 Danggui, 1 Baiying, 7 Longkui, 65 Danshen, 5 Shemei, 29 Banzhilian, and 15 Yujin active ingredients. After removing duplicates, a total of 134 active ingredients remained. Through target screening using "RelatedTargets" in TCMSP and "Swiss Target Prediction," a total of 290 target proteins were obtained after standardizing and removing duplicates using the UniProt database.

**Table 6:** Active ingredients (TCMSP)

molID	Compound	OB	DL	Herb
MOL000211	Mairin	55.38	0.78	Huangqi
MOL000239	Jaranol	50.83	0.29	Huangqi
MOL000296	Hederagenin	36.91	0.75	Huangqi
MOL000033	(3S,8S,9S,10R,13R,14S,17R)-10,13-dimethyl-17-[(2R,5S)-5-propan-2-yloctan-2-yl]-2,3,4,7,8,9,11,12,14,15,16,17-dodecahydro-1H-cyclopenta[a]phenanthren-3-ol	36.23	0.78	Huangqi
MOL000354	Isorhamnetin	49.6	0.31	Huangqi
MOL000371	3,9-di-O-methylnissolin	53.74	0.48	Huangqi
MOL000374	5'-hydroxyiso-muronulatol-2',5'-di-O-glucoside	41.72	0.69	Huangqi
MOL000378	7-O-methylisomucronulatol	74.69	0.3	Huangqi
MOL000379	9,10-dimethoxypterocarpan-3-O-β-D-glucoside	36.74	0.92	Huangqi
MOL000380	(6aR,11aR)-9,10-dimethoxy-6a,11a-dihydro-6H-benzofurano[3,2-c]chromen-3-ol	64.26	0.42	Huangqi
MOL000387	Bifendate	31.1	0.67	Huangqi
MOL000392	Formononetin	69.67	0.21	Huangqi
MOL000398	Isoflavanone	109.99	0.3	Huangqi
MOL000417	Calycosin	47.75	0.24	Huangqi
MOL000422	Kaempferol	41.88	0.24	Huangqi
MOL000433	FA	68.96	0.71	Huangqi
MOL000438	(3R)-3-(2-hydroxy-3,4-dimethoxyphenyl)chroman-7-ol	67.67	0.26	Huangqi
MOL000439	Isomucronulatol-7,2'-di-O-glucosiole	49.28	0.62	Huangqi
MOL000442	1,7-Dihydroxy-3,9-dimethoxy pterocarpene	39.05	0.48	Huangqi
MOL000098	Quercetin	46.43	0.28	Huangqi
MOL000358	Beta-sitosterol	36.91	0.75	Danggui
MOL000449	Stigmasterol	43.83	0.76	Danggui
MOL006859	Volon	35.42	0.63	Baiying/Baimaoteng
MOL002058	40957–99-1	57.2	0.62	Longkui
MOL002773	Beta-carotene	37.18	0.58	Longkui
MOL000359	Sitosterol	36.91	0.75	Longkui
MOL000546	Diosgenin	80.88	0.81	Longkui
MOL007356	Solanocapsine	52.94	0.67	Longkui
MOL000953	CLR	37.87	0.68	Longkui
MOL001601	1,2,5,6-tetrahydrotanshinone	38.75	0.36	Danshen
MOL001659	Poriferasterol	43.83	0.76	Danshen
MOL001771	Poriferast-5-en-3beta-ol	36.91	0.75	Danshen
MOL001942	Isoimperatorin	45.46	0.23	Danshen
MOL002222	Sugiol	36.11	0.28	Danshen
MOL002651	Dehydrotanshinone II A	43.76	0.4	Danshen
MOL002776	Baicalin	40.12	0.75	Danshen
MOL000569	Digallate	61.85	0.26	Danshen
MOL000006	Luteolin	36.16	0.25	Danshen
MOL006824	α-amyrin	39.51	0.76	Danshen
MOL007036	5,6-dihydroxy-7-isopropyl-1,1-dimethyl-2,3-dihydrophenanthren-4-one	33.77	0.29	Danshen
MOL007041	2-isopropyl-8-methylphenanthrene-3,4-dione	40.86	0.23	Danshen
MOL007045	3α-hydroxytanshinoneIIa	44.93	0.44	Danshen
MOL007048	(E)-3-[2-(3,4-dihydroxyphenyl)-7-hydroxy-benzofuran-4-yl]acrylic acid	48.24	0.31	Danshen
MOL007049	4-methylenemiltirone	34.35	0.23	Danshen
MOL007050	2-(4-hydroxy-3-methoxyphenyl)-5-(3-hydroxypropyl)-7-methoxy-3-benzofurancarboxaldehyde	62.78	0.4	Danshen
MOL007051	6-o-syringyl-8-o-acetyl shanzhiside methyl ester	46.69	0.71	Danshen
MOL007058	Formyltanshinone	73.44	0.42	Danshen
MOL007059	3-beta-Hydroxymethyllenetanshiquinone	32.16	0.41	Danshen
MOL007061	Methylenetanshinquinone	37.07	0.36	Danshen
MOL007063	Przewalskin a	37.11	0.65	Danshen
MOL007064	Przewalskin b	110.32	0.44	Danshen
MOL007068	Przewaquinone B	62.24	0.41	Danshen
MOL007069	Przewaquinone c	55.74	0.4	Danshen
MOL007070	(6S,7R)-6,7-dihydroxy-1,6-dimethyl-8,9-dihydro-7H-naphtho[8,7-g]benzofuran-10,11-dione	41.31	0.45	Danshen
MOL007071	Przewaquinone f	40.31	0.46	Danshen
MOL007077	Sclareol	43.67	0.21	Danshen
MOL007079	Tanshinaldehyde	52.47	0.45	Danshen
MOL007081	Danshenol B	57.95	0.56	Danshen
MOL007082	Danshenol A	56.97	0.52	Danshen
MOL007085	Salvilenone	30.38	0.38	Danshen
MOL007088	Cryptotanshinone	52.34	0.4	Danshen
MOL007093	Dan-shexinkum d	38.88	0.55	Danshen
MOL007094	Danshenspiroketallactone	50.43	0.31	Danshen
MOL007098	Deoxyneocryptotanshinone	49.4	0.29	Danshen
MOL007100	Dihydrotanshinlactone	38.68	0.32	Danshen
MOL007101	DihydrotanshinoneI	45.04	0.36	Danshen
MOL007105	Epidanshenspiroketallactone	68.27	0.31	Danshen
MOL007107	C09092	36.07	0.25	Danshen
MOL007108	Isocryptotanshi-none	54.98	0.39	Danshen
MOL007111	Isotanshinone II	49.92	0.4	Danshen
MOL007115	Manool	45.04	0.2	Danshen
MOL007118	Microstegiol	39.61	0.28	Danshen
MOL007119	Miltionone I	49.68	0.32	Danshen
MOL007120	Miltionone II	71.03	0.44	Danshen
MOL007121	Miltipolone	36.56	0.37	Danshen
MOL007122	Miltirone	38.76	0.25	Danshen
MOL007123	Miltirone II	44.95	0.24	Danshen
MOL007124	Neocryptotanshinone ii	39.46	0.23	Danshen
MOL007125	Neocryptotanshinone	52.49	0.32	Danshen
MOL007127	1-methyl-8,9-dihydro-7H-naphtho[5,6-g]benzofuran-6,10,11-trione	34.72	0.37	Danshen
MOL007130	Prolithospermic acid	64.37	0.31	Danshen
MOL007132	(2R)-3-(3,4-dihydroxyphenyl)-2-[(Z)-3-(3,4-dihydroxyphenyl)acryloyl]oxy-propionic acid	109.38	0.35	Danshen
MOL007140	(Z)-3-[2-[(E)-2-(3,4-dihydroxyphenyl)vinyl]-3,4-dihydroxy-phenyl]acrylic acid	88.54	0.26	Danshen
MOL007141	Salvianolic acid g	45.56	0.61	Danshen
MOL007142	Salvianolic acid j	43.38	0.72	Danshen
MOL007143	Salvilenone I	32.43	0.23	Danshen
MOL007145	Salviolone	31.72	0.24	Danshen
MOL007149	NSC 122421	34.49	0.28	Danshen
MOL007150	(6S)-6-hydroxy-1-methyl-6-methylol-8,9-dihydro-7H-naphtho[8,7-g]benzofuran-10,11-quinone	75.39	0.46	Danshen
MOL007151	Tanshindiol B	42.67	0.45	Danshen
MOL007152	Przewaquinone E	42.85	0.45	Danshen
MOL007154	Tanshinone iia	49.89	0.4	Danshen
MOL007155	(6S)-6-(hydroxymethyl)-1,6-dimethyl-8,9-dihydro-7H-naphtho[8,7-g]benzofuran-10,11-dione	65.26	0.45	Danshen
MOL007156	Tanshinone VI	45.64	0.3	Danshen
MOL001040	(2R)-5,7-dihydroxy-2-(4-hydroxyphenyl)chroman-4-one	42.36	0.21	Banzhilian
MOL012245	5,7,4’-trihydroxy-6-methoxyflavanone	36.63	0.27	Banzhilian
MOL012246	5,7,4’-trihydroxy-8-methoxyflavanone	74.24	0.26	Banzhilian
MOL012248	5-hydroxy-7,8-dimethoxy-2-(4-methoxyphenyl)chromone	65.82	0.33	Banzhilian
MOL012250	7-hydroxy-5,8-dimethoxy-2-phenyl-chromone	43.72	0.25	Banzhilian
MOL012251	Chrysin-5-methylether	37.27	0.2	Banzhilian
MOL012252	9,19-cyclolanost-24-en-3-ol	38.69	0.78	Banzhilian
MOL012254	Campesterol	37.58	0.71	Banzhilian
MOL012266	Rivularin	37.94	0.37	Banzhilian
MOL001973	Sitosteryl acetate	40.39	0.85	Banzhilian
MOL012269	Stigmasta-5,22-dien-3-ol-acetate	46.44	0.86	Banzhilian
MOL012270	Stigmastan-3,5,22-triene	45.03	0.71	Banzhilian
MOL000173	Wogonin	30.68	0.23	Banzhilian
MOL001735	Dinatin	30.97	0.27	Banzhilian
MOL001755	24-Ethylcholest-4-en-3-one	36.08	0.76	Banzhilian
MOL002714	Baicalein	33.52	0.21	Banzhilian
MOL002719	6-Hydroxynaringenin	33.23	0.24	Banzhilian
MOL002915	Salvigenin	49.07	0.33	Banzhilian
MOL000351	Rhamnazin	47.14	0.34	Banzhilian
MOL005190	Eriodictyol	71.79	0.24	Banzhilian
MOL005869	Daucostero_qt	36.91	0.75	Banzhilian
MOL008206	Moslosooflavone	44.09	0.25	Banzhilian
MOL004241	Curcolactone	51.51	0.2	Yujin
MOL004244	(4aR,5R,8R,8aR)-5,8-dihydroxy-3,5,8a-trimethyl-6,7,8,9-tetrahydro-4aH-benzo[f]benzofuran-4-one	59.52	0.2	Yujin
MOL004253	Curcumenolactone C	39.7	0.19	Yujin
MOL004260	(E)-1,7-Diphenyl-3-hydroxy-1-hepten-5-one	64.66	0.18	Yujin
MOL004263	(E)-5-Hydroxy-7-(4-hydroxyphenyl)-1-phenyl-1-heptene	46.9	0.19	Yujin
MOL004291	Oxycurcumenol	67.06	0.18	Yujin
MOL004305	Zedoalactone A	111.43	0.19	Yujin
MOL004306	Zedoalactone B	103.59	0.22	Yujin
MOL004309	zedoalactone E	85.16	0.19	Yujin
MOL004311	Zedoarolide A	87.97	0.3	Yujin
MOL004313	Zedoarolide B	135.56	0.21	Yujin
MOL004316	1,7-Diphenyl-3-acetoxy-6(E)-hepten	48.47	0.22	Yujin
MOL004328	Naringenin	59.29	0.21	Yujin

**Table 7:** Active ingredients (BATMAN)

PubChem CID	Compound	Molecular Formula	Molecular Weight(g/mol)	Herb
11850	Dulcitol	C_6_H_14_O_6_	182.17	Shemei
5316973	Ducheside A	C_20_H_16_O_12_	448.3	Shemei
442079	Ponalactone A	C_19_H_22_O_6_	346.4	Shemei
9930064	Roseoside	C_19_H_30_O_8_	386.4	Shemei
21122581	Rosamultin	C_36_H_58_O_10_	650.8	Shemei

### Target screening related to EGFR-TKI

**Fig. 5:**
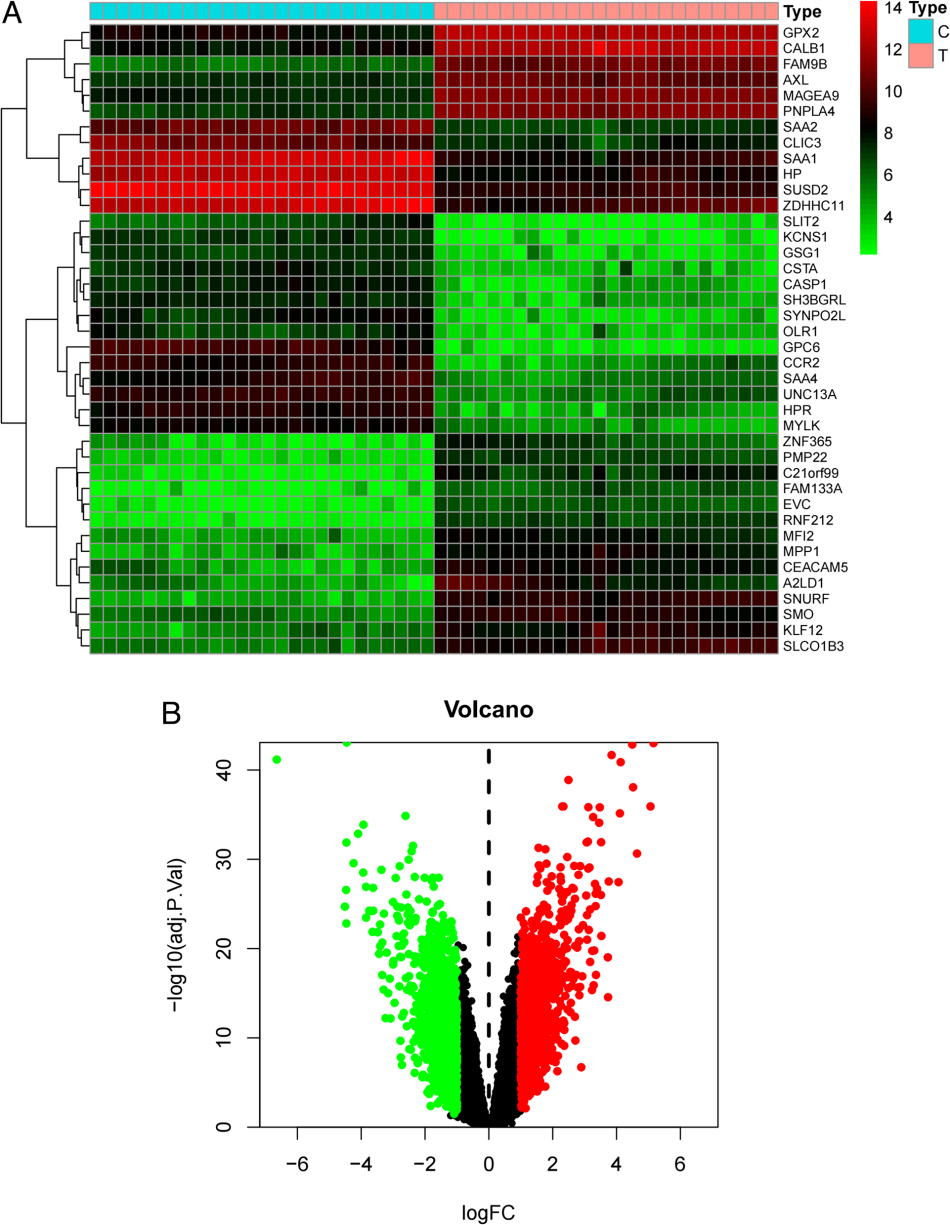
**A** Heatmap illustrating the positive and negative regulation of differentially expressed genes. **B** Volcano plot of differentially expressed genes between two cell line groups. Red represents upregulated genes, and green represents downregulated genes

Resistance data in GSE34228 was collected, which included sensitive and resistant cell lines to gefitinib. After *P* value filtering, a total of 976 upregulated genes and 1059 downregulated genes were obtained (Fig. 5). The heatmap illustrates the positive and negative regulation of differentially expressed genes, while the volcano plot shows the differentially expressed genes between the two cell line groups.

### Integration of common targets between ZLJT and EGFR-TKI resistance

By using R4.3.0 software, the intersection of effective ingredients in the ZLJT formulation and targets related to EGFR-TKI resistance was obtained. A total of 39 common targets were identified, indicating that the effective ingredients in ZLJT can exert their effects on TKI resistance through targets such as IL-6, FOS, STAT3, ERBB2, and EGF.

### "ZLJT-compound-target-disease" Network for delaying LUAD resistance

**Fig. 6:**
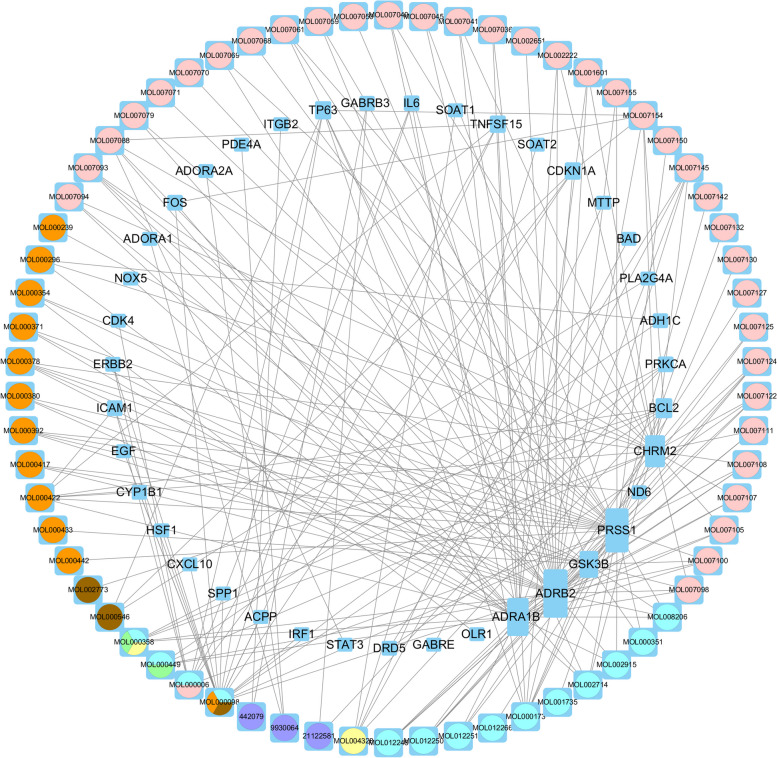
"ZLJT-compound-target-disease" network. A link represents the interaction between a compound and a target node. The small circle indicates target proteins. The large circle indicates active compounds; orange represents Huangqi, green represents Danggui, red represents Baiying, brown represents Longkui, pink represents Danshen, purple represents Shemei, blue represents Banzhilian, and yellow represents Yujin

The "ZLJT-compound-target-disease" network was constructed using the active compounds of ZLJT and the target genes associated with LUAD-TKI resistance, as shown in Fig. 6. The network consists of 39 gene nodes, 66 intersecting compounds, and 105 edges. The square-shaped blue nodes represent the target genes, with larger areas indicating more connections to other nodes. The circular nodes represent the effective compounds of the drug; orange represents Huangqi, green represents Danggui, red represents Baiying, brown represents Longkui, pink represents Danshen, purple represents Shemei, blue represents Banzhilian, and yellow represents Yujin.

### PPI network degree analysis

**Fig. 7:**
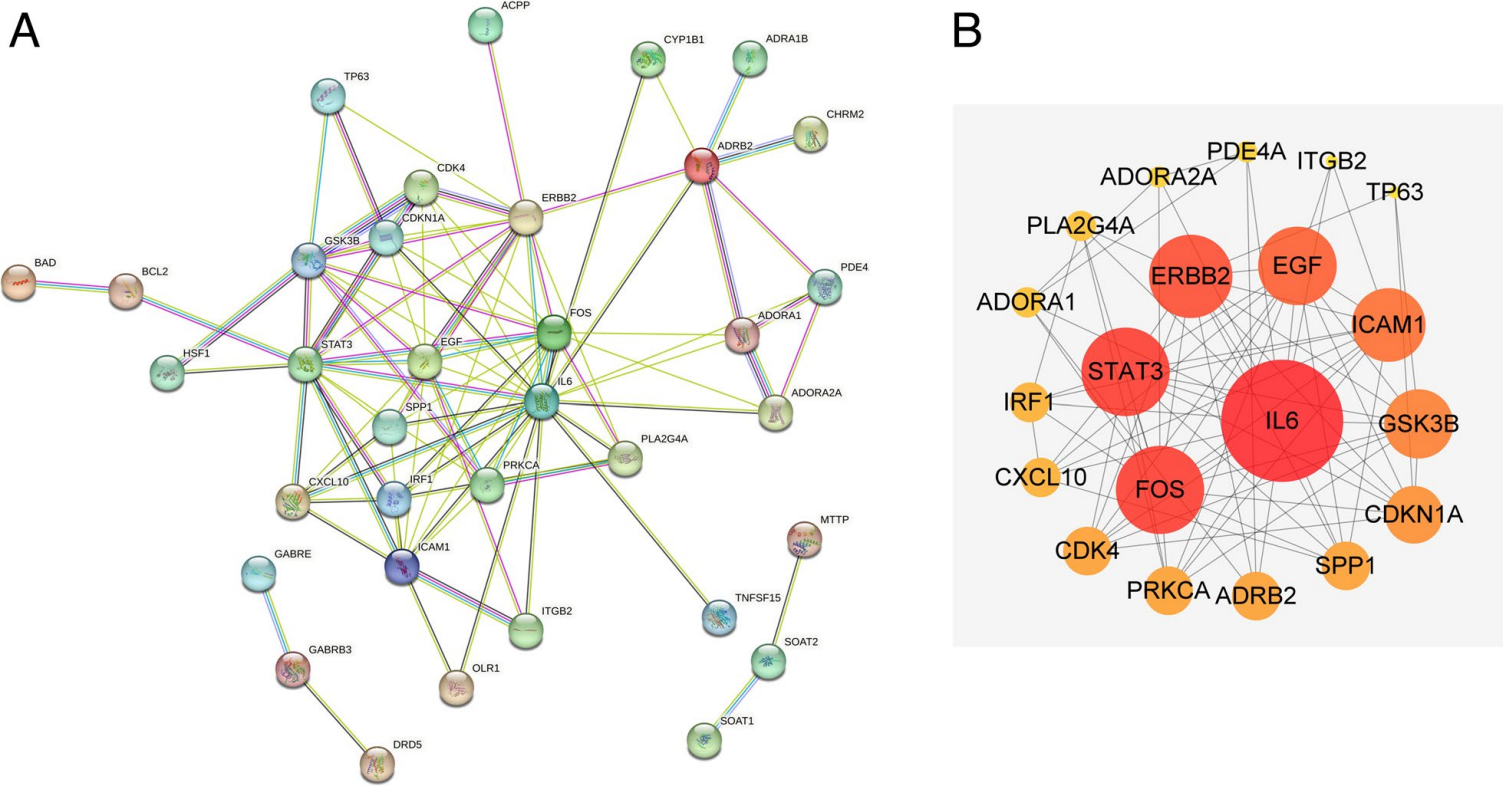
**A** The STRING PPI website of common targets. **B** Twenty core genes filtered by degree values

The 39 intersecting genes were uploaded to the STRING website, resulting in a PPI network graph (Fig. 7A). The PPI graph was then uploaded to Cytoscape 3.9.0 software for filtering based on the degree values, resulting in 20 core genes (Fig. 7B). These core genes include IL-6, FOS, STAT3, ERBB2, EGF, ICAM1, GSK3B, CDKN1A, SPP1, ADRB2, PRKCA, CDK4, CXCL10, IRF1, ADORA1, PLA2G4A, ADORA2A, PDE4A, ITGB2, and TP63, which are the key targets of ZLJT in delaying drug resistance.

### Bioinformatics analysis

**Fig. 8:**
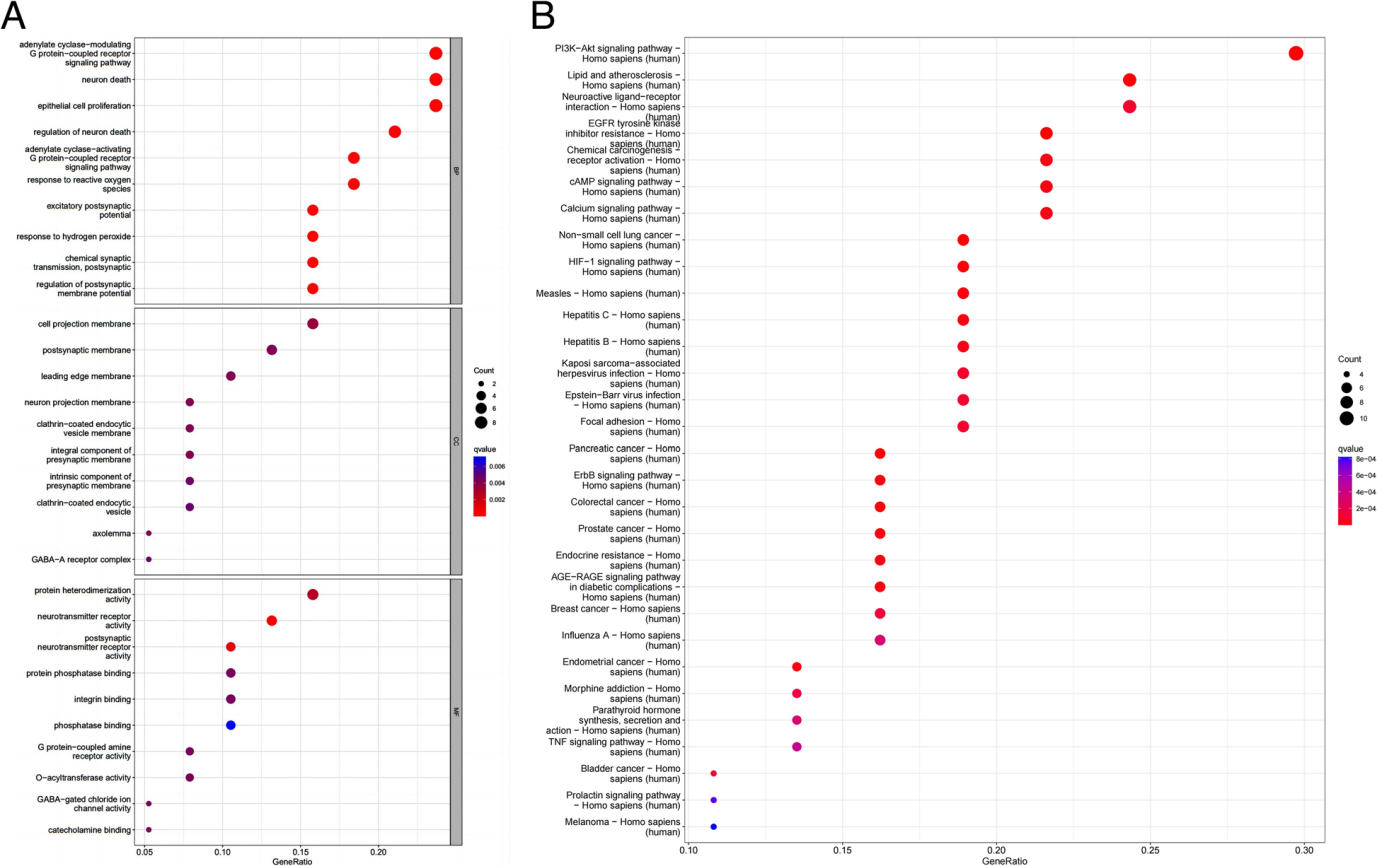
**A** The top 10 GO terms of hub genes. **B** The top 30 enriched pathways of hub genes

**Fig. 9:**
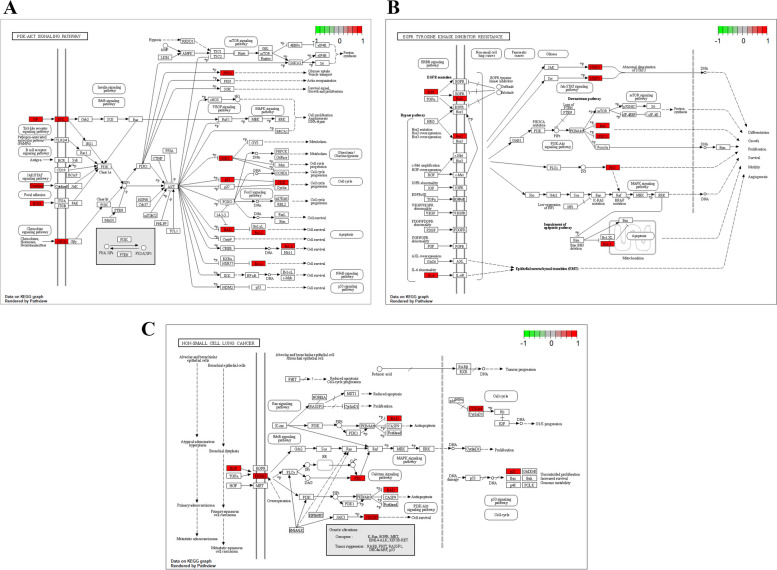
The important target genes were mainly distributed in the PI3K-AKT **A** and EGFR-TKI resistance **B** and NSCLC **C** signaling pathways

GO functional enrichment analysis of 214 common targets was performed using R4.3.0 software. The analysis yielded a total of 1,362 entries (*q* < 0.05), including 1,178 BP. The top three BP were adenylate cyclase-modulating G protein-coupled receptor signaling pathway, epithelial cell proliferation, and neuron death signaling pathway. In terms of CC, the top ones were cell projection membrane, postsynaptic membrane, and leading edge membrane. Additionally, there were 124 MF identified, such as protein heterodimerization activity, amide binding, and DNA-binding transcription. Bubble plots were created using the top 10 results for BP, CC, and MF (Fig. 8A). KEGG pathway enrichment analysis revealed 115 entries (*q* < 0.05), the top 30 enriched pathways were plotted in a bubble map (Fig. 8B). The PI3K-Akt (Fig. 9) signaling pathway played a crucial role in ZLJT resistance against LUAD cells. Furthermore, this pathway was also important in ZLJT resistance against lipid and atherosclerosis, neuroactive ligand-receptor interaction, and chemical carcinogenesis receptor activation signaling pathway.

### Molecular docking simulation

**Fig. 10:**
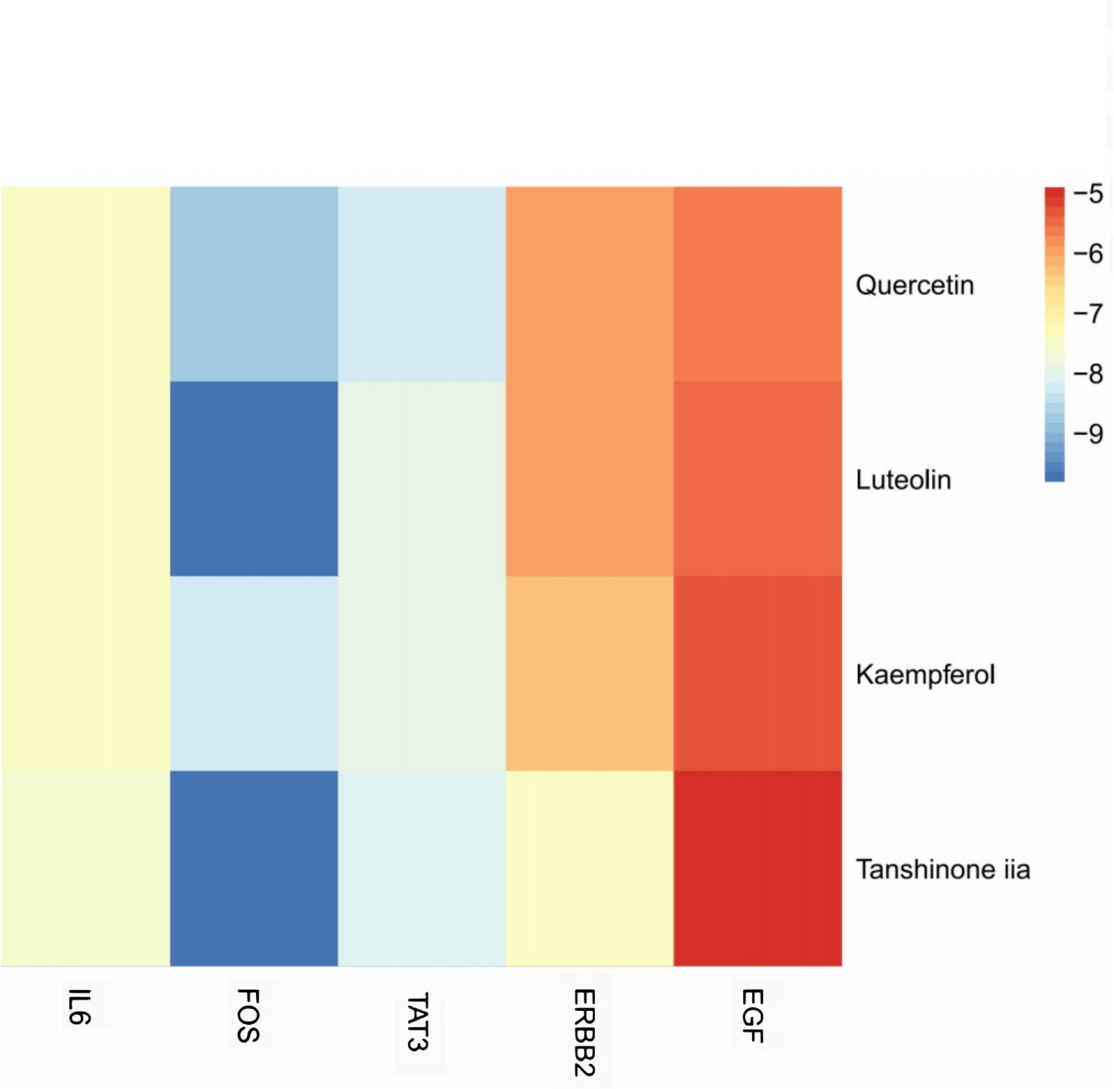
The results of the docking binding affinities

**Fig. 11:**
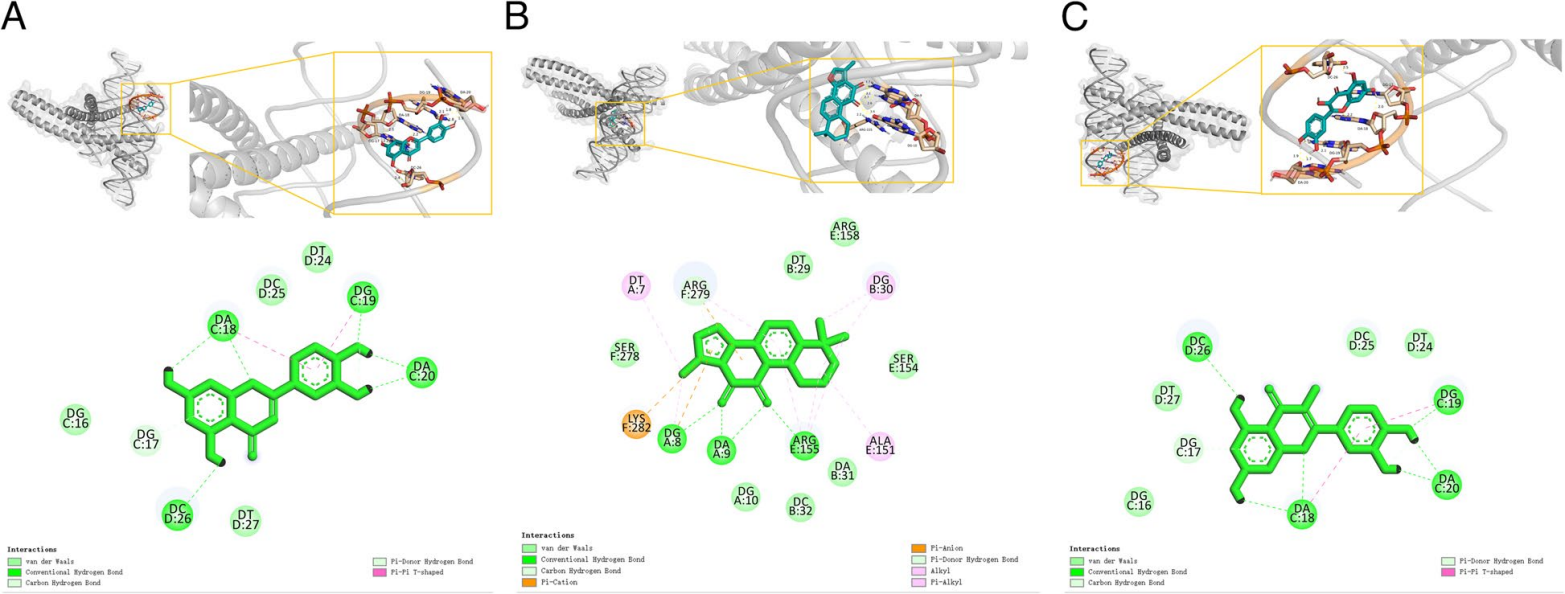
| Visualization of the binding models. **A** Luteolin and FOS (-9.8 kcal/mol). **B** Tanshinone IIA and FOS (-9.8 kcal/mol). **C** Quercetin and FOS (-8.7 kcal/mol)

Molecular docking simulations were performed between the top 4 active compounds from ZLJT and the top 5 core target proteins based on their degree values. The results of the docking binding affinities are presented in Fig. 10. The binding energy results of molecular docking between small molecules and large molecules are generally classified into three standards. A binding energy < -4.25 kcal/mol indicates a certain binding capability between the molecule and the target. A binding energy < -5.0 kcal/mol indicates a stronger binding capability, while a binding energy < -7.0 kcal/mol indicates a very strong binding capability. The magnitude of the binding energy can demonstrate whether there is binding capability between the small and large molecules. In addition to evaluating the binding energy, another criterion commonly used is the number of hydrogen bonds. It is generally considered that the formation of two or more hydrogen bonds indicates the formation of a stable conformation between large and small molecules. Among the active compounds that showed good performance in docking scores and binding modes with the 5 core target proteins (IL-6, FOS, STAT3, ERBB2, and EGF), luteolin, tanshinone IIA, and quercetin stood out. These compounds interacted with key amino acid residues, such as DA-20, DA-18, DG-19, DG-17, DC-26, DA-9, DG-10, and ARG-155. Luteolin contains multiple hydrophobic moieties, such as benzene rings and fused benzene rings, that can interact with other hydrophobic molecules, thus affecting their biological activity. In biological systems, luteolin and tanshinone IIA can interact with other molecules through hydrophobic interactions, thereby influencing their function and activity. Hydrophobic interactions can affect the interaction between tanshinone IIA and membrane proteins, enzymes, and other biological molecules, thereby affecting their biological effects and pharmacological properties. The hydrophobic moieties of quercetin can interact with other hydrophobic molecules, such as lipid bilayers and proteins, affecting cellular signal transduction, metabolism, enzyme activity, etc. Additionally, the hydrophobic moieties of quercetin can interact with hydrophobic amino acids in the cell membrane, influencing the stability and permeability of the cell membrane. Moreover, luteolin, tanshinone IIA, and quercetin showed a good match with the pocket of the FOS protein, and each part could interact well with the groove of the pocket. In summary, luteolin, tanshinone IIA, and quercetin exhibited the best binding affinity and docking scores with the FOS protein target among the active compounds. Other active compounds also formed hydrogen bonds with the amino acids at the binding site of the 5 target proteins and showed good compatibility with the protein targets. These compounds are key components of ZLJT in inhibiting late-stage LUAD resistance. Finally, the complexes formed by the docking of the active compounds with the protein targets were visualized using PyMOL 2.2.0 software, and the binding models with good binding affinity and high compatibility were selected for display. The visualization of the binding models is shown in Fig. 11.

## Discussion

In recent years, with the continuous advancement and clinical application of small molecule inhibitors targeting the EGFR, the progression-free survival (PFS) of patients with advanced NSCLC has significantly improved [27]. Studies have shown that TKIs have remarkable efficacy in the 5-year survival rate of advanced lung cancer [28]. EGFR-mutant NSCLC is more dependent on EGFR signaling than wild-type NSCLC [29]. EGFR-TKIs have been developed and proven effective in patients diagnosed with EGFR-mutant NSCLC. However, the development of acquired resistance to EGFR-TKIs is inevitable in these cases, despite the introduction of second- and third-generation EGFR-TKIs in an attempt to overcome this acquired resistance [30].

The mechanisms underlying the development of acquired resistance are not fully understood, but one of the most common mechanisms involves the secondary T790M mutation in the affected cancer gene, which leads to structural changes in the ATP-binding pocket and reduced affinity for TKI binding [31]. Additionally, other genetic alterations can drive the emergence of bypass clones, such as the activation of kinases., such as MET, IGF1R, BRAF, HER2, AXL, and FGFR, or changes in downstream pathway components, such as MAPK1 amplification, PIK3CA activation, or loss of PTEN or NF1. These alterations allow for the bypass of the requirement of EGFR in maintaining downstream ERK1/2 and AKT pathway activation, leading to acquired resistance [32, 33]. Studies have observed that the loss of PTEN and activating mutations in PI3K result in the constitutive activation of AKT, leading to the development of resistance [34].

In the clinical study involving 89 patients, it was observed that EGFR-mutant LUAD patients who received combination therapy of TKIs and ZLJT exhibited a significant extension in the time to acquired resistance compared to the TKI-only group, with a prolongation of 3.27 months (*P* < 0.01). This indirectly contributed to an improvement in patient progression-free survival (PFS). Among the data from the two groups, there was a statistically significant difference in the onset of resistance with the use of first-generation TKIs (*P* < 0.05), but no statistically significant difference was observed between the second- and third-generation TKIs. In the analysis of immune-inflammatory markers, the *P* values were all greater than 0.05, suggesting the possibility of insufficient sample size as a potential confounding factor. Regarding safety analysis, there was no statistically significant difference between the two groups, indicating the feasibility of combining ZLJT with TKIs in terms of safety.

To further explore the mechanism by which ZLJT delays LUAD EGFR-TKIs resistance, we conducted network pharmacology analysis and molecular docking techniques. In this study, we first collected compounds of ZLJT from databases, such as TCMSP and BATMAN-TCM, through network pharmacology analysis. Subsequently, we constructed a "ZLJT-compound-target-disease" regulatory network and identified the main active ingredients of ZLJT that have anti-LUAD cell resistance and inhibit tumor cell growth and migration, including quercetin, luteolin, kaempferol, and tanshinone IIA. Quercetin, a natural flavonoid, is found in many fruits and vegetables [35]. Related cell experiments have shown that quercetin can prevent lung cancer by scavenging free radicals, altering signal transduction pathways, inducing cell apoptosis, inhibiting phase I enzymes responsible for activating carcinogens, and inducing phase II enzymes responsible for detoxifying carcinogens [36, 37]. Clinical and pharmacological studies have demonstrated that quercetin possesses antioxidant and anti-inflammatory properties and exerts antitumor activity in the prevention of tumor progression by modulating oxidative and inflammatory networks [38]. An in vivo study found that luteolin inhibits NSCLC migration by controlling the actin cytoskeleton and inducing the migration-related proteins RAC1, CDC42, and RHOA [39]. Furthermore, luteolin has been shown to inhibit lung cancer cell proliferation by blocking LIMK1 and its related signaling pathways, leading to cell cycle arrest and apoptosis [40]. Luteolin exerts anticancer effects by inducing endoplasmic reticulum (ER) stress in a p53-independent manner and promoting autophagy in HEP3B (p53-null) cells, thereby enhancing cell viability [41].

It has been reported that kaempferol enhances anticancer potency when used in combination with other anticancer drugs. When combined with quercetin, kaempferol significantly increases the anticancer effect of quercetin by blocking its efflux. Moreover, kaempferol intake significantly enhances the cytotoxicity of cisplatin and increases the bioavailability of various anticancer drugs [42]. Tanshinone IIA exhibits anticancer activity in lung cancer cells. It induces autophagy and apoptosis and inhibits cell growth and migration by activating AMPK and inhibiting the PI3K/Akt signaling pathway [43]. Tanshinone IIA can sensitize cancer cells to TRAIL-induced apoptosis by upregulating DR5 or selectively activating PERK/ATF4 and inhibiting STAT3 [44]. In summary, the active ingredients in ZLJT exert their anticancer resistance effects by altering signal transduction pathways, blocking the tumor cell cycle, inhibiting tumor activity, enhancing cell viability, and improving the bioavailability of combination drugs.

PPI network analysis revealed that IL-6, FOS, STAT3, ErbB2, EGF, and other targets had high degree values and were the core targets of ZLJT in delaying LUAD resistance. IL-6 is involved in multiple physiological and pathological processes, such as cell proliferation, inflammation, and stress. Cancer-related inflammation significantly affects cancer metastasis, clinical manifestations of cancer, and patients' tolerance to anticancer treatment [45]. Effective inhibition of IL-6-induced signaling and transcription activator 3 (STAT3) phosphorylation can significantly downregulate the invasive ability of cancer cells [46]. It has been found that A431-V tumors resistant to vascular endothelial growth factor (VEGF) inhibitors secrete more IL-6 and exhibit higher levels of phosphorylated STAT3. Additionally, blocking IL-6 signaling on tumor cells can overcome this resistance [47]. In classic treatment for liver cancer, sorafenib can reverse resistance by blocking the IL-6 and AKT pathways [48]. Therefore, the IL-6/STAT3 signaling pathway is involved in the process of tumor cell resistance. FOS is a proto-oncogene associated with cell proliferation. FOS gene expression is activated by the combined action of extracellular nucleotides and growth factors. FOS has been shown to be a regulator of cell proliferation, differentiation, and transformation [49]. It has been reported that FOS plays a driving role in the development of lung cancer, breast cancer, and liver cancer and is involved in resistance to multiple drugs [50]. STAT3 is a transcription factor that, upon activation by growth factors and cytokines, participates in signal transduction and transcriptional activation. STAT3 is involved in the regulation of cell differentiation, proliferation, and survival in various malignancies and is associated with angiogenesis and immune dysfunction in tumor development [51]. Feedback activation of STAT3 plays a prominent role in mediating resistance to a wide range of targeted cancer therapies and chemotherapy and is considered an important target in anticancer drug resistance treatment [52]. ErbB2 is a member of the ErbB receptor tyrosine kinase family and plays a critical role in cell growth and proliferation signaling pathways. Amplification or overexpression of ErbB2 has been observed in 30% of breast cancer patients, driving cell transformation and cancer development [53]. In LUAD, ErbB2 has been identified as one of the driver genes [3], and its inhibition can attenuate tumor progression and cell invasion. Expression profiling analysis in human lung tumors has confirmed the importance of the ErbB2/Akt/ELF3 signaling pathway as a prognostic biomarker and therapeutic drug target for lung cancer treatment [53]. EGF plays a significant role in tumor cell proliferation and signal transduction. EGF activates EGFR signaling and promotes lung cancer cell proliferation, invasion, and metastasis. In solid tumors, such as non-small cell lung cancer, breast cancer, and esophageal cancer, the EGFR family is an important target in anticancer therapy [54]. In summary, ZLJT delayed EGFR-TKIs resistance may be closely related to IL-6, FOS, STAT3, ErbB2, EGF and other targets.

GO functional enrichment analysis revealed that the biological process of ZLJT to delay LUAD resistance mainly involves the adenylate cyclase-modulating G protein-coupled receptor signaling pathway, which plays a crucial role in various cancers, including lung adenocarcinoma. This pathway can contribute to cancer cell proliferation, invasion, and metastasis, making it a potential target for lung adenocarcinoma treatment. Additionally, the process involves epithelial cell proliferation since lung adenocarcinomas typically originate from epithelial tissues of the lungs. Understanding the mechanism of epithelial cell proliferation can provide valuable insights into the biology of lung adenocarcinoma. Moreover, neuron death may be associated with the effects of neurons in lung adenocarcinoma. Abnormalities in the neuron death signaling pathway could be linked to the tumor microenvironment and treatment response, thus influencing the development of cancer treatment strategies and pathological analysis. In terms of CC, the cell projection membrane may play a role in the invasion and migration process of lung adenocarcinoma cells. Investigating this process can enhance our understanding of the metastatic mechanisms of cancer cells and potentially improve therapeutic strategies. If lung adenocarcinoma occurs near the nervous system, the pathway associated with the postsynaptic membrane may be affected, which could have implications for studying specific subtypes of lung adenocarcinomas and making therapeutic choices. Furthermore, leading edge membranes are often involved in the process of cell migration and infiltration, which is crucial for characterizing tumor aggressiveness and metastasis in lung adenocarcinomas. Lastly, the molecular functions mainly include protein heterodimerization activity, an important component in signaling pathways. Understanding these activities can provide insights into protein interactions associated with lung adenocarcinoma. While amide binding may not be directly critical in the specific study of lung adenocarcinoma, it still holds biological significance. Additionally, DNA-binding transcription plays a crucial role in regulating gene expression, which is closely linked to the development and treatment response of lung adenocarcinoma.

Through KEGG enrichment analysis, the PI3K-Akt signaling pathway was identified as the top core node targeted by ZLJT in combating LUAD cell resistance. As an important signaling pathway, the PI3K-Akt signaling cascade is involved in the release of various proinflammatory cytokines, such as NF-κB, IL-6, and IL-17 [55]. Inflammation and cancer are fundamentally linked in the processes of development, invasion, and metastasis. Components of cancer-related inflammation can downregulate the activity of cytolytic enzymes, influencing tumor progression. These components include chemokines, prostaglandins, and cytokines [56]. Once activated by Akt, these cytokines play a role in tumor progression [57]. Therefore, ZLJT may exert its inhibitory effects on LUAD cell resistance by targeting the PI3K-Akt signaling pathway and its downstream targets. The pathway diagrams below provide a visual representation of the interactions between ZLJT and its core targets.

In addition, based on the results of KEGG pathway enrichment analysis, it is evident that ZLJT may play a role in delaying drug resistance in LUAD through several key pathways. Among these pathways, the lipid and atherosclerosis pathway is closely associated with tumor drug resistance. Tumors in harsh environments like hypoxia may increase the expression of growth factors and cytokines to bypass the drug target and generate potential drug resistance [58]. Additionally, tumor cells can synthesize exogenous lipids through uptake and activate endogenous lipids to promote tumor progression [59]. ZLJT may intervene in drug resistance in lung adenocarcinoma by affecting pathways related to lipid metabolism.Another significant pathway is the neuroactive ligand-receptor interaction pathway, which involves a set of neuroreceptors that may play a role in environmental information processing and signaling molecule interactions. This pathway may have a key role in the development and molecular specificity of lung cancer [60]. Imbalances in neuroactive ligand-receptor interaction may increase the risk of lung cancer. ZLJT may modulate the biology of lung adenocarcinoma, including drug resistance, by affecting this pathway [61]. Furthermore, ZLJT is closely linked to the chemical carcinogenesis receptor activation pathway, which involves DNA damage and repair. This pathway plays a role in tumor progression and chemoresistance after DNA damage. Disruptions in DNA repair can directly lead to the persistence of mutations with cellular oncogenic effects [62]. ZLJT may regulate the biological characteristics of lung adenocarcinoma, including drug resistance, by affecting the DNA damage and repair-related pathways to intervene in drug resistance in LUAD cells.The discovery of these pathway enrichments provides important clues for studying drug resistance in LUAD cells and may help to develop more effective therapeutic strategies.

The enrichment results suggest that ZLJT may affect EGFR tyrosine kinase inhibitor resistance and signaling pathways in non-small cell lung cancer. These findings provide strong support for the role of ZLJT in inhibiting drug resistance and delaying tumor progression in LUAD.

Molecular docking validation showed that luteolin, tanshinone IIA, and quercetin exhibited the strongest binding affinity and docking scores with the FOS target protein. Their binding affinities were -9.8, -9.8, and -8.7 kcal/mol, respectively. This indicates that active ingredients, such as luteolin, tanshinone IIA, and quercetin, in ZLJT play a critical role in inhibiting late-stage LUAD resistance.

However, further studies and experimental validation are needed to gain a deeper understanding of its mechanism and potential for clinical application.This study has certain limitations. Firstly, it only used the TCMSP and BATMAN-TCM tools for screening active ingredients of the drug, which may introduce bias in exploring the drug's active ingredients. To improve the level of evidence for drug ingredients, mass spectrometry or experimental studies can be conducted. Secondly, since the purpose of this article is to clinically retrospectively evaluate the effect of ZLJT combined with EGFR-TKI in delaying drug resistance in advanced lung adenocarcinoma, there is a lack of experimental verification of pathway research. Future studies should include relevant cell experiments or animal experiments to complete the evidence chain. Lastly, there were still limitations such as a single center and a small number of samples after screening, leading to a certain degree of bias in the results. Additionally, it is worth further considering and exploring how to better reduce interference factors in clinical experiments to ensure the scientific nature of the experiments.

## Conclusion

In summary, ZLJT has potential antitumor progression effects. In EGFR gene mutation-positive LUAD patients, the combination of EGFR-TKIs and ZLJT may have a role in delaying acquired resistance. Active ingredients, such as quercetin, luteolin, kaempferol, and tanshinone IIA can modulate signaling pathways related to NSCLC-EGFR cell resistance, lipids and atherosclerosis, chemical carcinogenesis receptor activation, and neuroactive ligand‒receptor interaction. They act on targets such as IL-6, FOS, STAT3, ERBB2, and EGF, exerting effects on delaying tumor cell resistance and inhibiting tumor cell invasion and metastasis. ZLJT may inhibit LUAD cell EGFR-TKIs resistance by altering signal transduction pathways, blocking the tumor cell cycle, suppressing tumor activity, enhancing cell vitality, and improving the bioavailability of coadministered drugs. Molecular docking simulations further validate the spatial and energy matching between the targets and active ingredients, providing theoretical evidence for the ability of ZLJT core components to delay LUAD resistance. This study provides insights for further mechanistic research and the clinical application of combating LUAD resistance. Therefore, more cell and animal experimental verification is still needed in the future to address the inherent limitations of this study.

## Data Availability

The original contributions presented in the study are included in the article/Supplementary Materials. Further inquiries can be directed to the corresponding authors.

## Abbreviations

LUAD: Lung adenocarcinoma
EGFR-TKI: Epidermal Growth Factor Receptor-tyrosine Kinase Inhibitor
ZLJT: ZiLongJin Tablet
AST: Aspartate Aminotransferase
ALT: Alanine Aminotransferase
CREA: Creatinine
TBIL: Total Bilirubin
ALB: Albumin
BUN: Blood Urea Nitrogen
NLR: Neutrophil-to-lymphocyte Ratio
PLR: Platelet-to-lymphocyte Ratio
TCMSP: Traditional Chinese Medicine Systems Pharmacology Database and Analysis Platform
BATMAN-TCM: A Bioinformatics Analysis Tool for Molecular mechanism of Traditional Chinese Medicine
OB: Oral bioavailability
DL: Drug likeness
GEO: Gene Expression Omnibus
DEGs: Differentially Expressed Genes
PPI: Protein‒protein Interaction
GO: Gene Ontology
NSCLC: Non-small cell lung carcinoma
NCCN: National Comprehensive Cancer Network
MF: Molecular Function
BP: Biological Process
CC: Cell Component
IL-6: Interleukin- 6
STAT3: Signal transducer and activator of transcription 3
ErbB2: HER2, Human Epidermal Growth Factor Receptor-2
